# Biomimetic Approach to Designing Trust-Based Robot-to-Human Object Handover in a Collaborative Assembly Task

**DOI:** 10.3390/biomimetics11010014

**Published:** 2025-12-27

**Authors:** S. M. Mizanoor Rahman

**Affiliations:** Department of Mechanical Engineering, The Pennsylvania State University, 120 Ridge View Drive, Dunmore, PA 18512, USA; mrahman@psu.edu

**Keywords:** human–robot collaboration, human–robot interaction, object handover, handover biomechanics, trust, bio-inspired robot motion planning, kinematic redundancy, assembly task

## Abstract

We presented a biomimetic approach to designing robot-to-human handover of objects in a collaborative assembly task. We developed a human–robot hybrid cell where a human and a robot collaborated with each other to perform the assembly operations of a product in a flexible manufacturing setup. Firstly, we investigated human psychology and biomechanics (kinetics and kinematics) for human-to-robot handover of an object in the human–robot collaborative set-up in three separate experimental conditions: (i) human possessed high trust in the robot, (ii) human possessed moderate trust in the robot, and (iii) human possessed low trust in the robot. The results showed that human psychology was significantly impacted by human trust in the robot, which also impacted the biomechanics of human-to-robot handover, i.e., human hand movement slowed down, the angle between human hand and robot arm increased (formed a braced handover configuration), and human grip forces increased if human trust in the robot decreased, and vice versa. Secondly, being inspired by those empirical results related to human psychology and biomechanics, we proposed a novel robot-to-human object handover mechanism (strategy). According to the novel handover mechanism, the robot varied its handover configurations and motions through kinematic redundancy with the aim of reducing potential impulse forces on the human body through the object during the handover when robot trust in the human was low. We implemented the proposed robot-to-human handover mechanism in the human–robot collaborative assembly task in the hybrid cell. The experimental evaluation results showed significant improvements in human–robot interaction (HRI) in terms of transparency, naturalness, engagement, cooperation, cognitive workload, and human trust in the robot, and in overall performance in terms of handover safety, handover success rate, and assembly efficiency. The results can help design and develop human–robot handover mechanisms for human–robot collaborative tasks in various applications such as industrial manufacturing and manipulation, medical surgery, warehouse, transport, logistics, construction, machine shops, goods delivery, etc.

## 1. Introduction

### 1.1. Human–Robot Collaboration in Industrial Environment

Robots in traditional industrial scenarios are commonly separated from humans by placing robots within specially designed work cells or restricting the coexistence of robots with humans using different cages or barriers [1]. This policy can be adopted partly due to safety for humans and partly due to the notion that the abilities of humans and the capabilities of robots are not complementary [2]. However, this notion has significantly modified in recent years with the understanding that the abilities of humans and the capabilities of robots can be complementary if they can be designed and implemented appropriately [3,4]. Recent advancements in collaborative robots (e.g., Dobot CR5 [5], UR5 [6]) have made it possible for humans to safely work in the workspace of robots. As a result, there is growing interest in the field ‘Human–Robot Collaboration (HRC)’ where human co-workers and robots can share the same workspace, communicate with each other in real-time, and collaborate on the same tasks for a shared goal [7,8]. In recently emerged concepts of HRC, the respective strengths and abilities of humans (e.g., perception or sensing, adaptability, decision-making even in uncertain situations) and of robots (e.g., accuracy, repeatability, precision) can be used to mitigate the common limitations of robots and of humans in a complementary manner [9,10].

### 1.2. Human–Robot Collaboration in Manufacturing

The manufacturing industry can contribute significantly to the GDP of nations that want to maintain a strong economy [11]. Assembly operation in the manufacturing of most products is a stage in which the highest percentage of value can be added [12,13]. Usually, manual assembly operations in manufacturing may be a burden to the human workforce, may be inefficient, and may adversely impact workers’ health and safety (though this may not always be true) [14]. On the contrary, industrial robots and related industrial automation systems employed for assembly operations can be very expensive, and these are inflexible in most cases [15]. As a result, it may appear that the strengths and capabilities of robots and human workers can be made complementary to each other, and they can collaborate with each other to make assembly tasks more flexible and adaptable, and the collaborative assembly tasks may be more productive than cases when human–robot collaboration does not occur [16]. Such collaboration seems to be more beneficial for small-to-medium scale manufacturing processes because such processes may have more variations in the requirements of the tasks, workmanship, resources, equipment, and methods [17]. Therefore, there is growing emphasis and interest in HRC in assembly in industrial manufacturing where collaborative robots and human co-workers can share the same workspace and work environment, and they can work on the same tasks together [18,19]. Considering this possibility, HRC in assembly operations in industrial manufacturing has become an active area of research where researchers have addressed different aspects of HRC in assembly tasks [20,21,22]. However, systematic research efforts on HRC in assembly tasks addressing the needs of actual industrial scenarios have not been reported extensively in the literature, except for a few preliminary initiatives [23,24,25].

### 1.3. Handover of Payloads in Human–Robot Collaboration

An important aspect of HRC is handovers or handoffs of payloads between robots and human co-workers [26]. For example, an assistant-type collaborative robot may carry a tool or a part and hand it over to a human co-worker when the human co-worker needs it [27]. Applications of such handover operations can range from human patient care in hospitals or therapy centers to astronaut Extra-Vehicular Activities (EVAs) [28,29,30]. Handovers or handoffs can be bidirectional, i.e., the payload may go from a human to a robot [31], or it may go from a robot to a human during collaboration [32]. HRC through handovers has emerged as an active area of research and development, with significant contributions made toward advancements in design and performance of handover operations [28,29,30]. As the literature shows, human-to-robot handovers are conventional [31], but robot-to-human handovers are a new concept, which is gaining importance and attention [32,33,34]. In [35], human–robot handovers were designed and developed, being inspired by the social cues in human–human handovers. A study focused on identifying the best poses for comfortable robot-to-human handovers [36]. Motion planning strategies for robot-to-human handovers were investigated in [37,38]. A fuzzy rule-based control strategy for robot-to-human handovers was investigated in [39]. Handover operations between humanoid robots and astronauts were presented in [40], and so forth. It is realized that successful and efficient handovers of payloads or objects (parts) between human co-workers and collaborative robots are very important and relevant for HRC in assembly tasks in manufacturing because assembly tasks can include robot-to-human handover operations of objects (e.g., assembly parts, tools) that can benefit the overall assembly tasks [41]. However, the literature does not show significant efforts toward developing robot-to-human handover operations in conjunction with assembly tasks in industries, except for a few preliminary initiatives, e.g., in [42,43].

### 1.4. User-Friendliness and Safety in Handovers

Adjustments between robots and human co-workers and safety issues in handovers in collaborative tasks are very critical and important for human co-workers for long-term work relationships and sustainable collaboration between robots and humans through handover operations [44]. Safety issues are more critical for robot-to-human handovers than human-to-robot handovers because, unlike a human co-worker, a collaborative robot cannot adjust its handover configuration and motion easily in changing or uncertain task situations [45]. However, research efforts on robot-to-human handovers in HRC tasks, especially in assembly tasks that address human co-workers’ adjustment and safety issues, are not so mature yet [44,46].

### 1.5. Trust-Safety Tradeoffs in Handovers

In the state-of-the-art HRC tasks involving handovers [35,36,37,38,39,40], the concept of trust was ignored. Trust can be defined as the willingness of a human co-worker to rely on or to believe in the cooperation or assistance provided by the robot counterpart for collaborative tasks (e.g., handovers, co-manipulation) [47]. A high level of trust or at least a satisfactory level of trust of a human co-worker in a collaborating robot is necessary because the human co-worker may not show interest in collaborating with the robot if the human cannot trust the robot [47]. Trust is considered a vital issue not only in human–robot collaborative systems, but also in various other agent-based systems. For example, a fuzzy logic approach was deployed to model customer trust in vendors in transactions in e-commerce [48], quantitative analysis of controls of security based on dynamic trust modeling and evaluation in cloud environment was conducted [49], trust-related conditions of possible routes in selecting low risk routes for data transmission in wireless networks were investigated [50], and so forth. Human trust in collaborating robots has been reported in a few recent HRC studies, e.g., [51,52]. However, the current HRC research involving trust is neither related to manufacturing nor handovers, except for the preliminary research conducted on trust-based HRC in assembly in manufacturing operations [53,54]. Furthermore, well-developed techniques to model human trust in robots and methods to measure trust in real time are not available in the literature. Apart from very limited studies, e.g., [55,56], very few study in formally modeling a robot co-worker’s trust in a collaborating human have been conducted. Robot trust in a human co-worker is a novel idea, and it is believed that robot trust in a human co-worker may increase transparency and predictability of robots’ inherent states, behaviors, and actions to a human, which may improve human–robot team fluency for handovers in a collaborative task [55,56,57]. The status of collaborating robot trust in its human co-worker may reduce the human’s cognitive workload, and thus the human may devote more cognitive resources to handovers instead of worrying about or explaining the actions and behaviors of the robot in a collaborative setup [55,56]. However, robot trust in a collaborating human co-worker, especially in payload handovers in assembly in manufacturing operations, has not received much priority [58].

Trust in human–human handover operations was studied in [59], which reveals that a human co-worker tries to adjust their postures approaching a handover based on their trust level in the handover partner (e.g., the counterpart in the collaborative task). For example, when a human is unsure of their partner’s behavior, the human may try to configure their postures to minimize the effects of prospective impulse forces in the direction of the handover with an aim of mitigating the effects of their partner crashing into him/her [59]. Similar findings reported in [60] showed that human hand trajectories and applied load and grip forces in robot-human manipulation tasks changed when the human experienced uncertainty, doubt, or an unusual situation regarding the co-manipulated objects. Being inspired by these human psychological behaviors, it is expected that a similar strategy applied to a robot could result in safe and compliant robot-to-human handovers if robot trust in the human partner for the handover could be modeled and estimated in real time [58]. Such a trust-triggered biomimetic handover motion planning strategy for robots utilizing its kinematic redundancy may achieve the proposed safe and compliant handovers [58,61]. However, such a compliant biomimetic motion planning strategy for robot-to-human handovers triggered by robot trust in human co-workers exploiting the kinematic redundancy characteristics of the robot is not available in the literature, except for a few preliminary efforts, e.g., [42].

### 1.6. Objective and Summary of the Research

To address the research gaps identified above, the objective of this article was to develop a novel motion planning algorithm or strategy for a collaborative robot for robot-to-human handover of a payload in a human–robot collaborative assembly task based on the robot’s trust in the human co-worker, following biomimetic principles, exploiting the benefits of kinematic redundancy of the robot manipulator. An HRC assembly task was developed in a human–robot hybrid cell. In a biomimetic study, human handover behaviors in a human-to-robot handover task at different levels of human trust in the robot were investigated. Then, a computational model of robot trust in humans was derived, and a method to measure the trust in real-time was developed. Based on the computed value of robot trust in the human, the robot’s handover motion and configuration were adjusted by exploiting kinematic redundancy features of the robot to minimize potential impulse forces on the human co-worker through the payload during a robot-to-human handover operation associated with the HRC assembly task. The biomimetic and kinematically redundant handover motion planning strategy was to allow the robot to follow a cautious movement when its trust in the human would be low, which would help it enhance safety, and an aggressive movement when its trust would be high, which would help it enhance efficiency. In a robot-to-human handover experiment, it was justified how the combination of cautious and aggressive handover movements made the overall handover motion of the robot safe, compliant, human-friendly, and productive for the HRC task.

### 1.7. Organization of the Paper

The organization of the article is as follows: In Section 2, the materials and resources used for the research are presented. The materials and resources included the task context for the proposed human–robot collaboration and handover, design and development of the experimental (physical) human–robot system, robot-human trust model, and trust-triggered robot handover motion planning algorithm. Experimental methods, procedures, and results are presented in Section 3. A general discussion is presented in Section 4, and the conclusions and future work are presented in Section 5. Then, the acknowledgement and references are presented.

## 2. Materials and Resources

### 2.1. Context of the Task

We focused on a potential human–robot collaborative assembly task scenario observed in small-scale flexible manufacturing processes in different industries [53,54]. Small-scale industrial assembly processes may be more uncertain, unpredictable, and unstructured compared to large-scale industrial automation processes, and thus, the requirements of resources may be less determinable in advance due to frequent and sudden changes in processes and product parameters and requirements. Collaborative industrial robots can possess high precision and repeatability, but well-designed collaboration between collaborative robots and human co-workers can incorporate extra flexibility into the assembly processes [62]. We considered such a collaborative task environment between a human co-worker and a collaborative robot to efficiently complete an assembly task in a human–robot hybrid cell [63,64]. We made the following assumptions about the selected task context:

- The assembly task could be segmented into a finite number of subtasks, and each subtask could be assigned to the human co-worker, the collaborative robot, or both based on an optimum subtask allocation strategy [24]. Each subtask might include the manipulation, acquisition, retrieval, attachment, or storage of parts, components, instruments, or tools in the assembly process that were to be performed or used in a pre-determined sequence [24]. Once the subtask allocation strategy and procedure were determined, the agents (the human co-worker and the collaborative robot) needed to perform the assigned subtasks sequentially while keeping pace with each other. The human co-worker used their dexterous (hand) skills to perform each subtask assigned to him/her. But the robot was enriched with necessary motion planning, sensing, and control schemes to perform each subtask assigned to it [24].
- The human–robot or robot–human payload handovers occurred during the human–robot collaborative assembly task. The payloads could be assembly materials or parts, equipment, tools, accessories, etc. The human–robot handover task could be bidirectional, which means that the robot could be an assistant robot and it handed the payload over to the human co-worker during the assembly task (the robot was the giver and the human co-worker was the receiver of the payload), and the opposite could also happen (the human was the giver and the robot was the receiver) [24,56].
- In the proposed human–robot collaborative assembly task, the same robot manipulator was used to collaborate with the human co-worker for both performing the assembly task and handing over one or multiple payloads to the human co-worker as needed for the assembly task. Therefore, the handover was designed as an irregular or need-based subtask within the collaborative assembly task. The robot gave the payload to the human during the handover process, i.e., it was a robot-to-human handover [34]. The regular assembly processes needed to pause temporarily while the robot was handing over the payload to the human; the assembly processes might resume once the handover process ended.
- Initially, the collaborative robot needed to pick the payload from a designated location on a table surface, grasp the payload stably, and hand it over to the human in the common workspace between the robot and the human for the collaborative assembly task [42].
- Finally, it was assumed that, in ideal conditions, the handover configuration and process were sufficiently structured, and the robot was enriched with a preplanned trajectory based on task-optimality for the handover operation [65]. However, such trajectory planning might be adjusted later based on the robot’s trust in humans during the handover operation.

### 2.2. Human–Robot Handover System Design and Development

**Figure 1:**
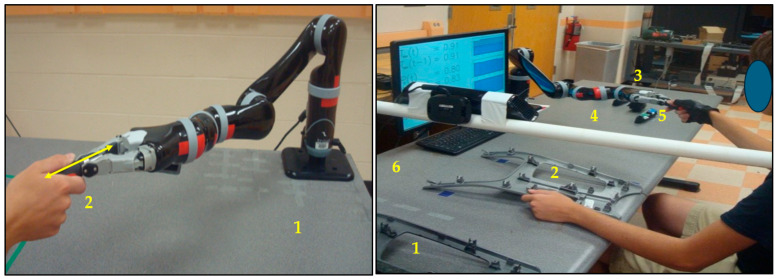
The human–robot object handover system (**left**), and the human–robot object handover system included in a human–robot collaborative assembly task (**right**).

A 6DOF Kinova MICO robotic arm (2-finger manipulator, weight 5.2 kg, max payload 1.3 kg, reach 0.7 m, DOF 6, power consumption 25 W) was fixed on a table, as Figure 1 (left) shows. It was programmed in such a way that it was able to pick an object from the table surface (from location 1) and hand it over to a human (robot-to-human handover) at location 2, or it could receive an object from a human at location 2 and put it on the table surface (human-to-robot handover) at location 1, as Figure 1 (left) shows. Such a human–robot handover operation was added to a human–robot collaborative assembly task, as Figure 1 (right) shows. In the collaborative assembly task, as Figure 1 (right) shows, different components of a center console of an automobile were initially placed on a table surface (the center console main frame was placed at location 1, and switch rows and I-drive components were placed at location 3). The objective was to assemble the components to build a center console for the automobile (at location 2). For this purpose, the entire task was divided into several subtasks, and each subtask was assigned to either the human or the robot based on an optimum subtask allocation strategy [24]. The sequence of the subtasks was decided based on the actual requirement of the assembly task. The sequence of each subtask and its assigned performer (either the human or the robot) was displayed in the computer monitor placed in front of the human. There was a sound system for the computer. The sound system also pronounced the sequence of each subtask so the human could clearly understand the subtask sequence. Based on the allocation of subtasks and their sequence, the robot manipulator picked the switch rows and I-drive components from location 3 of the table surface and manipulated them to the front of the human (placed them at location 4) following the predefined subtask sequence [24]. The human co-worker picked the main center console frame from location 1, manipulated it to location 2, took switch rows and I-drive components from location 4 as per the sequence, and assembled them manually at location 2 using a screwdriver. Then, the human dispatched the finished (assembled) product from location 2 to location 6 on the left side of the table.

The objects that were handed over during the assembly task could be additional assembly parts or components (accessories), assembly tools such as a new screwdriver, etc., located initially at location 3. The robot manipulator could pick the handover payload from location 3 and manipulate it to location 5 to hand it over to the human at location 5. The human co-worker could participate in the handover task in their sitting or standing position, depending on task conditions. The robot manipulator was controlled by a computer with a monitor. The motion control algorithm was coded in Python on the computer, and it communicated with the robot manipulator via ROS (Robot Operating System) [66]. The robot manipulator followed a task-optimal trajectory during its manipulation operations [24]. The human was asked to follow the shortest distance straight line trajectory during the manipulation of any object or component. Mock trials were arranged before actual experimental trials to train human subjects in understanding the workspaces of the human and of the robot. The above arrangements clearly differentiated between the human workspace and the robot workspace during the collaborative task and avoided any overlap between those two workspaces, which was essential to avoid collisions between the human and the robot during the collaboration due to potential overlaps between their trajectories.

### 2.3. Human–Robot and Robot–Human Bilateral Trust Computational Model

A human–robot and robot–human bidirectional trust computational model was required to reflect human and robot trust in the human–robot handover operation. As per the literature, most state-of-the-art trust computational models consider human trust in another human or in an artifact collaborating with the human, such as a robot, machine, automation device, etc. [67], instead of robot trust in humans, except for a few preliminary initiatives, e.g., [55,56]. If we consider human trust in a robot, then such trust can be influenced by different factors related to the robot, task, task environment, and other humans involved in the collaborative task [68]. Trust is always a perceptual issue, and a human can possess an actual perception of their trust in a robot. However, it is not possible to give the robot a similar perception of its trust in the human. From an analytical study, we know that robots’ faults (mistakes at work) and performance can be correlated with the trust of a human collaborator in the robot (in their robot counterpart) [69]. Lee and Moray proposed a regression model for identifying relevant factors of human trust in robots or automation [70]. They proposed a time-series computational model based on a robot’s faults and performance to compute the trust, as Equation (1) expresses, where t stands for time step, $\emptyset_{1}$, $\emptyset_{2}$, $A_{1}$, and $A_{2}$ stand for real-valued constants that are relevant to the human–robot system in question, and *q* stands for the random noise perturbations or uncertainties in trust computation [67,68,69,70].

(1) $$Trust\;\left(t\right)=A_{1}Performance\left(t\right)+A_{1}\emptyset_{1}\;Performance\left(t-1\right)+A_{2}Fault\left(t\right)+A_{2}\emptyset_{2}Fault\left(t-1\right)+q\left(t\right)$$

Trust may depend on many factors. However, Equation (1) considers agent (human, robot, automation, etc.) performance and fault factors to develop the trust computational model because these two factors (performance and faults) were found to influence trust the most [69]. Nonetheless, this model may not reflect the actual trust of a human, but it may help incorporate trust in the robot-human handover operation. Based on Equation (1), the computational model of human trust in the robot (denoted as $T_{H2R})$ can be derived as in Equation (2), where $P_{R}$ and $F_{R}$ stand for the reward scores for the robot for its performance and fault status respectively, and $b_{1},b_{2},\;c_{1},and\;c_{2}$ are constant coefficients with real values that can depend on the collaborative task itself, the human and the collaborative robot [24]. Being inspired by Equation (2), we may use a similar time-series model to compute the trust of the robot in its human counterpart (we denoted it as $T_{R2H})$, which is expressed in Equation (3), where $P_{H}$ and $F_{H}$ stand for the reward scores for the human co-worker for their performance and fault status, respectively, and $\varphi_{1},\varphi_{2},\;\omega_{1},\omega_{2}$ stand for constant coefficients with real values that can depend on the collaborative task itself, the human and the robot. Here, $q_{1}$ and $q_{2}$ stand for the random noise perturbations or uncertainties in trust computation for $T_{H2R}$ and $T_{R2H}$, respectively, *t* is the time step at which the computation is updated. $T_{H2R}$ might be updated at every discrete time step, *t,* based on the real-time or nearly real-time measures of $P_{R}$ and $F_{R}$ at every *t*. Similarly, $T_{R2H}$ could be updated at every discrete time step, *t*, based on the real-time or nearly real-time measures of $P_{H}$ and $F_{H}$ at every *t*. It is possible to normalize the values of $T_{H2R}$ and $T_{R2H}$ between 0 or 0% (no trust) and 1 or 100% (the maximum trust). It is also possible to consider distrust or over-trust of the robot in the human or of the human in the robot. However, we might ignore it herein, as the trust measures between 0 or 0% and 1 or 100% for the selected collaborative task might be sufficient to reflect human and robot trust in handover configuration and motions [24].

Many factors or variables may influence trust [67]. We considered only two factors to compute trust because those were the main factors influencing trust [68]. Consideration of more factors or variables in the trust models could enhance the accuracy of trust computations, but it might add complexity to the models and reduce model practicality and ease of real-world implementation of the models [67,68,69,70]. This is why the models were simplified into two-factor models to enhance their practicality. A tradeoff between model accuracy and complexity should be maintained when deciding on trust models from a practical perspective. The proposed trust models were deterministic models that could capture trust variations through capturing variations in performance and faults with respect to time. Variations in the constant coefficients could be considered to transform the deterministic models to probabilistic or stochastic models [67,68,69,70].

(2) $$T_{H2R}\left(t\right)=b_{1}P_{R}\left(t\right)+b_{2}P_{R}\left(t-1\right)+c_{1}F_{R}\left(t\right)+c_{2}F_{R}\left(t-1\right)+q_{1}\left(t\right)$$

(3) $$T_{R2H}\left(t\right)=\varphi_{1}P_{H}\left(t\right)+\varphi_{2}P_{H}\left(t-1\right)+\omega_{1}F_{H}\left(t\right)+\omega_{2}F_{H}\left(t-1\right)+q_{2}\;(t)\;$$

## 3. Methods and Results

### 3.1. Experiment 1: Taking Inspiration from Human Handover Behaviors

The objective of this experiment was to investigate how humans behaved when they handed over payloads to a collaborative robot at different levels of trust in the collaborative robot. The special interest was to learn human hand kinematics and kinetics at the time of object handover to a robot co-worker at different levels of human trust in the robot.

#### 3.1.1. Subjects

A power analysis was conducted. The details of the power analysis were as follows: continuous endpoint, two independent sample type studies, alpha value = 0.05, beta value = 0.1, and power value = 0.90. The power analysis with those particulars resulted in a sample size of 30. Based on this result, 32 human subjects (human factors, psychology, engineering students, and researchers) were recruited for this experiment. Out of the 32 recruited subjects, 16 subjects were male, and 16 were female. The mean age of the subjects (based on what they reported) was 23.68 years (STD =2.17). As part of the best ethical practice, written consent was collected from each subject regarding their timely participation in the experiment protocols. Appropriate ethical guidelines were followed, and we were aware of the safety, respect, and privacy of the human subjects participating in the experiment. Brief training and instructions on experimental procedures were provided to the subjects before they participated in the experiment.

#### 3.1.2. Experiment Procedures and Protocols

The human–robot collaborative system demonstrated in Figure 1 (right) was used for this experiment. In each trial, a human subject handed over a screwdriver to the robot. The robot received the screwdriver from the human’s hand (dominant hand) and placed it at a designated location on the table surface. The human and the robot performed such a human-to-robot payload handover 3 times consecutively. Human trust in the robot was computed following Equation (2) (the detailed procedures of trust computation in real-time were presented previously in [24]). Based on some practice trials using the subjects and the experimental system shown in Figure 1 (right), we first estimated the parameter values (values of the constant coefficients) necessary to compute human trust in the robot in real time based on the model derived in Equation (2). To do so, we used information on robot performance and faults observed in the practice trials to compute the constant coefficients of the computational trust model following the Autoregressive Moving Average Vector (ARMAV) model [71]. The estimated values of the constant coefficients are shown in Table 1.

The computed trust values were recorded for the 3 consecutive handovers, but the values were not disclosed to the human subject. The absolute velocity of the human hand was measured using an IMU (Inertial Measurement Unit) that the subject wore at their wrist. The angular position of the human hand (arm) with respect to the robot arm position was measured using the IMU. Two foil strain gauge-type force sensors were attached to the screwdriver handle to measure the grip forces that the human subject applied when handing over the screwdriver to the robot. At the end of the experiment, the human subject was asked to respond to a 7-point Likert scale (1 was the worst and 7 was the best response) to express their trust in the robot for the collaborative payload handover. Human response, human hand velocity, angular position, and grip forces were recorded for each trial. The same procedures were repeated for all other subjects, and each subject participated in the experiment separately. The subjects were selected randomly.

### 3.2. Results of Experiment 1

Table 2 results show an empirical relationship between subjectively assessed trust values and mathematically computed trust values of human co-workers in the collaborating robot. We see in Table 2 that when the subjects subjectively rated their trust in the robot as ‘High’ (between 5.50 and 7.0 on the Likert scale), the computed trust values for the trials involving those subjects were also high (between 0.8 and 1.0), though the computed trust values were not disclosed to the subjects during the experiment. Thus, the results demonstrated that the computed trust values correlated with the subjectively assessed trust values, which in turn proved the practicality and effectiveness of the trust computational model presented in Equation (2) [67,68,69,70].

The results in Table 3 show the mean human hand velocities, hand angular positions, and grip forces during object handover for different computed trust levels of humans in the robot. The results show that human psychology was significantly impacted by human trust in the robot, which also impacted the biomechanics of human-to-robot handover, i.e., human hand movement slowed down, the angle between human hand and robot arm increased (formed a brace-like configuration), and human grip forces increased for the situations when human trust in the robot was low, and vice versa. The results show that humans moved their hands with normal velocities, their hands were almost parallel to the robot arm, and the grip forces used to grip the object (payload) were not so high (it was the lowest) when human trust in the robot was high. It happened in high human trust in robot situation (when robot performance was high and robot faults/mistakes were low) because humans might assume that the robot was well-prepared, it was reliable and in good situations and it was capable of receiving the object from humans properly, and thus the humans did not need to take any extra caution or strategy to handover the object to the robot. However, in low trust situations (when robot performance was poor and its faults/mistakes were high), humans might think that the robot was not reliable and well-prepared to receive the object from the humans competently. As a result, humans might adopt some extra cautious strategies to avoid any unexpected situations (e.g., the object might fall during the handover, the robot arm might hit the human arm, etc.) during object handover. As part of such extra cautious strategies, humans might slow down their hand movement velocities, configure the handover in such a way that there was a significant angle between human hand and the robot arm (i.e., they formed braced configurations) and increase grip forces to protect the object from falling when handing the object over to the robot in low trust situation [42].

Table 3 also shows that human hand velocities, hand angular positions, and grip forces for the cases when humans handed over an object to the robot for the first time were almost the same as those for the low trust situations. It might happen because humans were unsure about their trust in the robot (i.e., trust did not grow up because humans did not have experience of working with the robot until that time), and as a result, humans followed the most cautious strategies (slowed down hand movement velocities, created braced handover configurations by increasing the angle between human hand and robot arm and increased grip forces) to tackle any unexpected situations to ensure a smooth and trouble-free human-to-robot handover of an object [72].

We analyzed each human subject’s dominant hand trajectories for handing over the payload (screwdriver) to the robot at different trust conditions: (a) high trust, (b) medium trust, and (c) low trust. The hand trajectories clearly showed that the human hesitated to hand over the payload to the robot; there were jerks (lack of smoothness) in the hand movement trajectories, and the human tried to take their hand back from the robot when their trust in the robot became low. Such hesitation was probably because the human thought that the robot was not prepared, and it was not reliable in situations where the robot did not perform well and/or made mistakes, which resulted in low human trust in the collaborating robot. Such results might also be responsible for causing reduced human hand velocity and braced handover configurations in low human trust situations, as exhibited in Table 3. However, the handover trajectories showed that each human subject used a very smooth hand trajectory to give the payload to the robot when human trust in the robot was high. The findings indicated that humans also became unprepared, irregular, unsure, or hesitant when giving the payload to the robot if they realized their reduced trust in the robot [59]. The unplanned hand trajectories could cause violent contact between the human hand and the robot end effector, which could create excessive and harmful impulse forces. The handover trajectory analysis results showed that in reduced human trust circumstances, the human needed to take an initiative to save the robot as well as themselves from the impulse forces by reducing prospective impulse forces through generating “braced-like” configurations of handover with slower (cautious) handover motion, which Table 3 justifies.

### 3.3. Experiment 2: Robot Handover Motion Planning Inspired by Human Handover Behaviors

#### 3.3.1. Bio-Inspired Handover Motion Planning Strategy for the Robot

**Figure 2:**
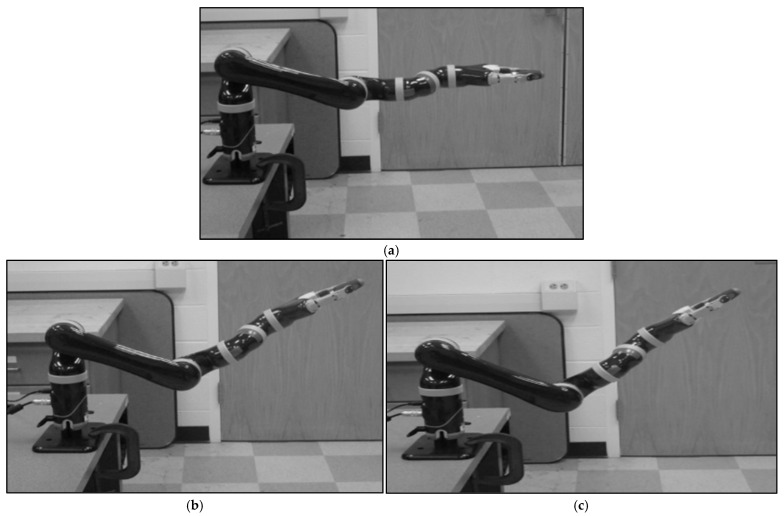
Robot-to-human object handover configuration of the robot when (**a**) the robot has high trust in the human, (**b**) when the robot has medium trust in the human (partly braced handover configuration), and (**c**) when the robot has low trust in the human (almost fully braced configuration).

Being inspired by the results of experiment 1, a novel trust-based handover motion planning strategy for the robot was proposed in this section. We assume that a real-time or nearly real-time measure of robot trust in its human co-worker was given. Then, the objective was to use the computed robot trust values to modify and adjust the handover behaviors of the robot, i.e., modify the robot’s motion and configuration during object handover [42]. We divided the handover motion planning of the robot into two different categories: (i) normal or default handover, and (ii) corrective or adjusted handover. The robot’s handover motion and configuration were considered normal or default (human hand was to be in parallel/co-linear with the robot end effector) when the robot’s trust in its human co-worker remained high, as shown in Figure 2a [42]. In this condition, the robot would follow the preplanned task-optimal handover trajectory, which would be safe for the human co-worker because the human might be in a well-planned situation and thus would be aware of potential impulse forces since the human would maintain high robot trust in them through their high performance and reduced faults during the handover. However, when robot trust in humans drops to below a pre-specified threshold, we might need to correct or adjust the robot’s handover motion trajectory based on the computed trust values, as shown in Figure 2b. As the figure shows, when robot trust in humans is reduced from high to medium, the robot would try to generate a partial braced handover configuration with reduced handover motion to mitigate the potential impulse forces generated during the handover. When robot trust in humans is low, it means that humans would not be in a good position to collaborate with the robot, i.e., humans would be unprepared for the collaboration. As a result, if the robot handed an object over to the human, the human might be unable to receive the object properly. It means, in the low robot trust in the human, the human might prove unreliable to receive the object. If the robot handed an object over to the human when the human was unprepared and unreliable, impulse or impact forces due to unplanned and unwanted relative motions between the human hand and the robot end effector via the object might be generated during the handover, and the forces might be exerted on the human hand via the object. Such impulse forces might be unsafe for the human’s occupational health and safety and for the successful completion of the handover task (the payload might drop off, or it might be damaged). As a result, the robot would generate a braced configuration with a reduced handover speed (cautious trajectory) to mitigate potential impulse forces, as Figure 2c illustrates [42].

A robot manipulator may have high accuracy and repeatability in motion planning, including handover motion planning. As a result, the robot can generate normal handover motion even when its trust in the collaborating human becomes low. However, we cannot directly control or adjust the handover motion of the human co-worker because the human’s handover motion planning is related to human psychology. As a result, to mitigate or minimize the effects of unwanted relative motion and resulting impulse forces for the condition when the robot’s trust in the human becomes low, we may adopt a novel handover motion planning strategy for the robot itself, as Figure 2 illustrates. The novel handover motion planning strategy for the robot may be such that the robot itself may take an initiative to control its own handover motion to minimize the effects of potential impulse forces resulting from relative motions (unexpected contact between the human hand and robot end effector through the object) when robot trust in the human becomes low [42]. To realize this novel handover motion planning strategy for the robot, we here adopt a two-fold approach: (i) the robot follows a ‘cautious’ handover motion, which is such that the robot slows down the handover motion, and (ii) the robot forms a ‘braced configuration’ when handing over an object to the human. It is assumed that the slow motion and the braced handover configuration can avoid or minimize potential collision and the resulting impulse forces in the direction of the human co-worker receiving an object (payload) from the robot during the handover operation when the robot’s trust in the human is low. This strategy may slightly reduce the efficiency of the handover task, but it may enhance overall safety and productivity of the assembly task involving object handover operations [73]. Taking inspiration from Table 3, the absolute velocities for the handover configurations in Figure 2a–c were empirically decided as 0.7, 0.6, and 0.4 of the robot arm’s maximum speed (1.0), respectively.

#### 3.3.2. Bio-Inspired Handover Motion Planning Algorithm for the Robot

**Figure 3:**
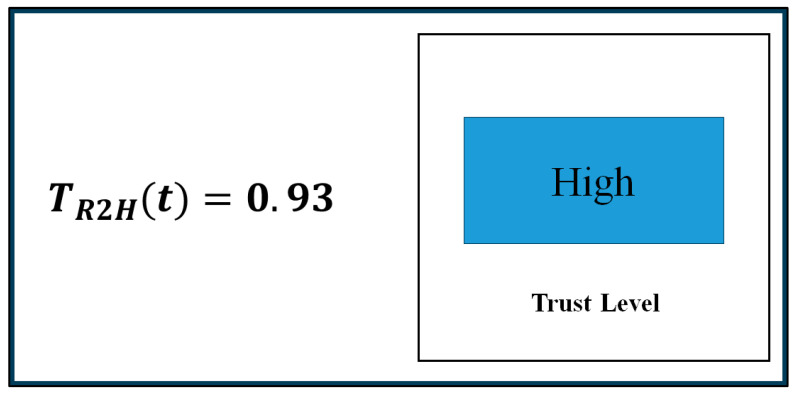
Illustrating the layout of the visual interface to display computed robot trust in humans on the computer screen and on the robot screen in real-time. The trust values and trust levels were updated at every *t* seconds.

**Figure 4:**
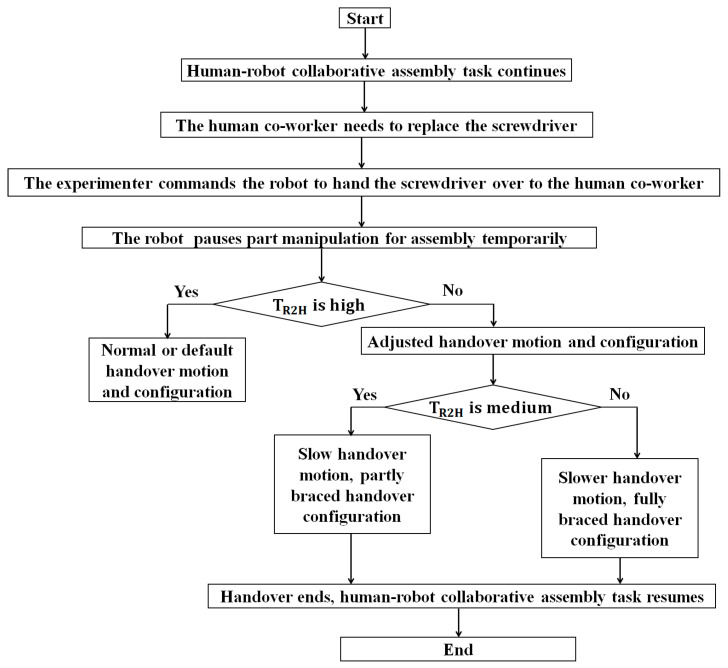
The trust-triggered (bioinspired or biomimetic) robot-to-human object (screwdriver) handover algorithm during human–robot collaborative assembly task for flexible manufacturing.

To realize the trust-based handover motion planning strategy for the robot introduced in Figure 2, we herein computed robot trust in the human in real-time following Equation (3). To do so, we estimated the values of the constant coefficients (trust model parameters) used to compute robot trust in the human co-worker in real time, following the same Autoregressive Moving Average Model (ARMAV) method [71], which we followed earlier for computing human trust in the robot. The values of the constant coefficients are shown in Table 4. The detailed procedures of trust computation in real-time were presented previously [24]. Once robot trust in the human was computed, we displayed the computed trust values in real time on the monitor of the computer used to control the robot and on the robot’s face screen, as Figure 3 illustrates. Based on the computed trust values, as Figure 3 illustrates, we designed a trust-triggered (bio-inspired) handover motion planning algorithm for the robot, as demonstrated in Figure 4, to realize the handover motion planning strategy proposed in Figure 2.

#### 3.3.3. Experimental Protocols and Procedures

The objective of this experiment was to evaluate the effectiveness of the bio-inspired robot-to-human object handover algorithm proposed in Figure 4 in a human–robot collaborative assembly task setup as introduced in Figure 1 (right). In this experiment, we used the same human subjects that we used in experiment 1. Each subject separately conducted the collaborative assembly task with the robot, and the assembly task also included a robot-to-human handover operation. There were two experimental protocols, as follows:

(i). The collaborative assembly task, including a robot-to-human handover operation, was executed, and the handover followed the handover configurations introduced in Figure 2 and the algorithm introduced in Figure 4, depending on computed robot trust values. We call this experimental protocol “Bio-inspired (trust-triggered) handover”.
(ii). The collaborative assembly task, including a robot-to-human payload handover operation, was executed, but the handover did not follow the algorithm introduced in Figure 4 (i.e., the robot used the default or normal handover motion and configuration as Figure 2a demonstrates. We call this experimental protocol “Default handover”.

For the assembly task associated with bio-inspired handover, human subjects and the robot performed the collaborative assembly, including a handover event, following Figure 1 (right). Each subject separately performed the assembly task with the robot based on the subtask allocation for 5 min, following the same procedures as described in Experiment 1. $T_{R2H}$ was measured and displayed on the screen in real time (see Figure 3). While the assembly operation was going on, the experimenter randomly decided a time during the assembly and asked the human subject to use a new screwdriver, replacing the existing screwdriver. The subject then sent a handover command to the robot by pushing the “Enter” key on the keyboard. The robot then stopped manipulating assembly parts and moved to pick the screwdriver from a corner of the table, manipulated it to the human, and handed it over to the human, as Figure 1 (right) demonstrates. The robot followed one of the three handover configurations (Figure 2a–c) with corresponding handover motions (normal/fast, slow, or very slow motion) based on the computed robot trust (value) in the human at that time step, according to the algorithm demonstrated in Figure 4. At the end of the handover trial, the assembly operation resumed. At the end of the assembly task, we evaluated the collaborative assembly task, including the handover operation, following a comprehensive evaluation scheme, as follows. Each subject took part in the experiment separately, and they were selected for the experiment randomly.

For the assembly task without an associated bio-inspired handover (default handover), the above procedures were repeated, and $T_{R2H}$ was measured and displayed in real time (Figure 3). However, the robot handed over the screwdriver to the human following a fixed (default) handover configuration and motion as illustrated in Figure 2a.

#### 3.3.4. Evaluation Scheme

**Figure 5:**
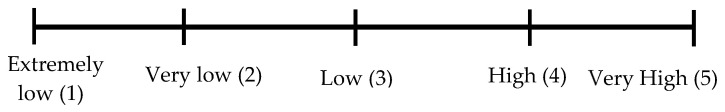
The Likert-type scale to assess pHRI.

We developed an evaluation scheme to evaluate each of the experiment protocols introduced above. The evaluation scheme included HRI (human–robot interaction) criteria and collaborative assembly performance criteria related to the KPI (key performance indicator) of the collaborative assembly [24]. The HRI criteria were categorized into pHRI (physical HRI) and cHRI (cognitive HRI) categories [74]. The pHRI criteria were subjectively assessed by each human subject for the entire assembly task (including the handover operation) using a Likert scale between 1 and 5 as shown in Figure 5, where score 5 indicated very high and score 1 indicated extremely low pHRI. We determined a set of pHRI criteria as explained in Table 5. We expressed cHRI in two criteria: (i) human subject’s trust in the robot, and (ii) human subject’s cognitive workload while collaborating with the robot for the collaborative assembly task, including the handover operation [42,46]. We used the same Likert scale as shown in Figure 5 to assess human trust. We followed the paper-based NASA TLX [75] for assessing the cognitive workload of the subjects while collaborating with the robot for the task.

We expressed the assembly performance in several criteria as follows: (i) Handover success rate ($\varepsilon_{hasr}$) was computed using the formula expressed in Equation (4), where $h_{f}$ stands for the number of failed handover trials and $h_{t}$ stands for the total number of handover trials. (ii) Safety in the handover ($\varepsilon_{sh}$) was computed using the formula expressed in Equation (5), where $h_{c}$ stands for the number of handover trials when human subjects experienced impulse forces, and $h_{t}$ stands for the total number of handover trials. (iii) Handover efficiency ($\lambda_{he}$) was computed using the formula expressed in Equation (6), where $T_{mtsh}$ stands for the mean targeted or standard time and $T_{mrh}$ stands for the mean recorded time for a handover trial. (iv) Assembly efficiency ($\lambda_{ae}$) was computed using the formula expressed in Equation (7), where $T_{mtsa}$ stands for the mean targeted or standard time for an assembly trial including handover and $T_{mra}$ stands for the mean recorded time for an assembly trial including handover. All criteria were evaluated through the experiment for each subject separately.

(4) $$\varepsilon_{hasr}=\left(1-\frac{h_{f}}{h_{t}}\right)\times 100\%\;$$

(5) $$\varepsilon_{sh}=\left(1-\frac{h_{c}}{h_{t}}\right)\times 100\%$$

(6) $$\lambda_{he}=\frac{T_{mtsh}}{T_{mrh}}\times 100\%$$

(7) $$\lambda_{ae}=\frac{T_{mtsa}}{T_{mra}}\times 100\%$$

In addition to the HRI criteria and assembly performance criteria, we measured the human and robot kinematics data for each assembly trial. An encoder-type position sensor was attached to the gripper of the robot manipulator to measure the absolute velocity of the robot arm’s last link (end effector) in each trial. A foil strain gauge-type force sensor was attached inside the robot end effector (gripper) to measure the grip force of the robot arm’s last link (end effector) in each trial.

#### 3.3.5. Experimental Results and Analyses

**Figure 6:**
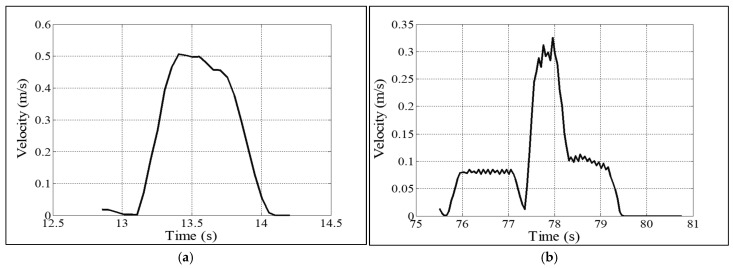
Sample absolute velocity profile of the robot arm’s last link (end effector) for (**a**) high trust, and (**b**) low trust situations.

We compared the absolute velocities of the robot’s last link (end effector) during the handover at high trust, medium trust, and low trust of the robot in the human. Figure 6 shows sample absolute velocity profiles of the robot arm’s last link (end effector) for the robot’s high-trust and low-trust situations. Table 6 compares the mean values of the robot arm’s end effector’s absolute velocities for different computed robot trust levels in the human. The results show that the robot used modified (braced type) handover configurations with reduced absolute velocities when the robot had medium and low trust in the human [42]. The reason might be that the robot needed to be more cautious in its handover motion planning when it realized that its trust in the human became medium to low. Table 6 also compares the mean values of the robot arm’s end effector’s grip forces for different computed robot trust levels in humans. The results show that the robot used modified (braced type) handover configurations with higher grip forces when the robot had medium and low trust in the humans. The reason might be that the robot needed to be more cautious to secure the object (payload) when it realized that its trust in humans became medium to low [42].

**Figure 7:**
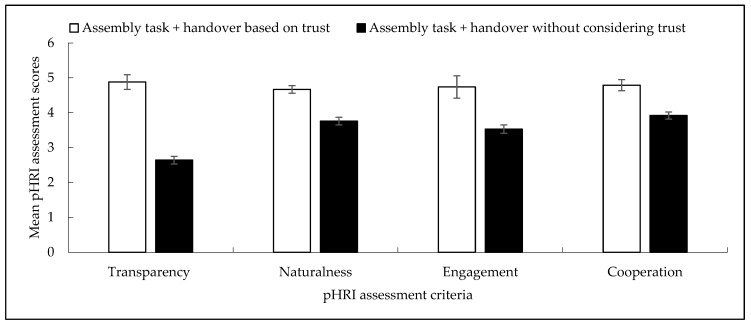
Comparison of pHRI perceived by human subjects between two different conditions related to trust (two different experimental conditions: trust-based handover vs. traditional handover).

Figure 7 compares the pHRI perceived by human subjects between two different experimental conditions: assembly task plus handover based on trust (“trust-based handover”) and assembly task plus handover without considering trust (“traditional handover”). The comparison results showed that the pHRI results for the trust-based handover were significantly better than those for the traditional handover. It is believed that the real-time display of trust and trust levels served as warning messages to the human collaborators and made the contextual information transparent to them. The change in robot handover configurations based on its trust in humans also acted as a high-class communication approach by the robot manipulator to convey the prevailing trust situations and potential consequences due to trust status to the humans [76], which might enhance transparency in the human–robot collaborative assembly and handover task. It is also believed that the prevailing transparency and the “cautious (slow)” handover configurations and kinematic strategies helped the human feel natural, which might enhance naturalness in the collaborative task perceived by the human co-workers. On the other hand, the real-time trust display (higher transparency) and adjustments in handover configurations helped the humans mentally and physically connect and attune with the robot manipulator, which might enhance the engagement level of the human with the robot [77]. We may also argue that the highly transparent, natural, and engaged collaboration between the human and the robot during the collaborative task resulted in the perception of more intense cooperation between the human and the robot for the trust-based handover condition over the traditional handover condition. Those were the most probable reasons behind the comparatively better perceived pHRI for the trust-based handover condition [42].

We conducted analysis of variances (ANOVAs) on each pHRI criterion (transparency, naturalness, engagement, cooperation, team fluency) separately. ANOVA results showed that variations in pHRI assessment results due to consideration of robot trust in robot handover configuration and kinematic adjustment were statistically significant (p<0.05 for each pHRI criterion, e.g., for transparency, F=4.84, p<0.05), which indicated that consideration of robot trust produced significantly differential effects on pHRI perceived by the subjects. On the other hand, variations between the subjects who participated in the study were not statistically significant (p>0.05 for each pHRI criterion, e.g., for transparency, F=3.73, p>0.05), which indicated that the results could be considered as the general results even though a comparatively small number of human subjects participated in the study [46].

**Figure 8:**
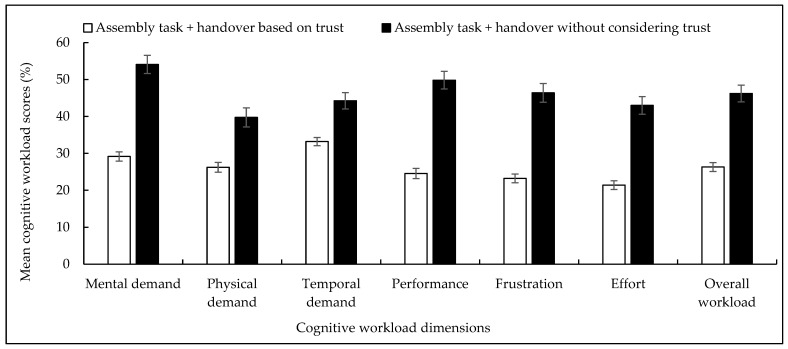
Assessment results in comparing the mean values of the six dimensions of the NASA TLX between trust-based and traditional handover strategies.

As we compared the cHRI assessment results, Table 7 shows a 43.09% reduction in the mean cognitive workload and a 27.70% increment in human trust (subjectively assessed) in the robot for the trust-based handover condition compared to the traditional handover condition. It is believed that the advantages in the pHRI results (especially the prevailing transparency in the performance and fault status of the human co-workers expressed through the computed trust values displayed on the screen) might reduce the cognitive workloads of humans. Figure 8 shows the comparison of the overall cognitive workloads between the two handover conditions. The figure also compares in detail the assessment results for each of the six dimensions of the NASA TLX between the trust-based and traditional handover conditions. We conducted ANOVAs on each of the cHRI criteria (workload and human trust) separately. ANOVA results showed that variations in the cHRI assessment results were statistically significant (p<0.05 for each criterion, e.g., for cognitive workload, F=42.07, p<0.05). The results showed the significance of the effects of consideration of robot trust in the handover design on cHRI assessment results for the human co-workers. For the ANOVA results, variations between the human subjects were not statistically significant (p>0.05 at each criterion, e.g., for cognitive workload, F=3.64, p>0.05). ANOVA results proved the generality of the cHRI results even though a comparatively small number of human subjects participated in the study [24].

The results in Table 8 show 30%, 30%, and 7.82% improvement in handover safety, overall handover success rate, and overall assembly efficiency, respectively, for the trust-based handover condition over the traditional handover condition. Table 9 compares the mean impulse (collision) forces between trust-based and traditional handover conditions. It is believed that the “cautious (slow)” handover kinematics (motion) and “braced-like” handover configurations of the robot’s end effector for comparatively low robot trust situations (medium and low trust situations) might reduce potential collisions and impulse forces during the handover operation. It is also believed that the reduction in the potential collisions and impulse forces might improve the safety for the trust-based handover over the traditional handover [46]. It also seemed to be rational that enhanced safety and pHRI might improve the overall handover success rate for the trust-based handover condition over the traditional handover condition [78].

We see that the results in Table 8 show 0.90% less handover efficiency for the trust-based handover condition compared to the traditional handover condition. It is assumed that it might happen due to the requirement of larger handover time for the cautious (slower) handover motions that we set for different cautious handover strategies demonstrated in Figure 2b,c for the low trust situations. However, the mean overall assembly efficiency involving the trust-based handover operation was clearly higher than the mean overall assembly efficiency involving the traditional handover operation, as we can see in Table 8. This phenomenon can be explained by considering the overall performance of the human–robot collaborative task. It is believed that the “cautious” handover motions for low trust situations reduced the handover efficiency a little bit due to the reduced absolute speeds we set for low trust conditions, as demonstrated in Figure 2b,c. However, the advantages gained in pHRI, cognitive workload, and safety made up the loss of efficiency reduction due to the “cautious” handover operations and thus helped increase the overall efficiency of the entire assembly task involving the trust-based handover condition [42].

Moreover, the handover operations followed the “cautious” motions only when the robot’s trust in humans was low. It is believed that trust display and trust-based warning messages (Figure 3), superior pHRI, and reduced cognitive workload enhanced human performance and reduced human faults in the collaborative task, which helped maintain a high $T_{R2H}$. Consequently, the robot in most cases followed the default handover trajectory (Figure 2a) as reflected in the results presented in Table 9. The results in Table 9 show that the robot did not need to follow the cautious handover motions in 80% of the assembly trials. The table also shows that the robot followed the medium cautious handover trajectory (Figure 2b) in 15% assembly trials, and the very cautious handover trajectory in only 5% assembly trials. As the results show, the prevailing high-trust situations did not necessitate cautious handover trajectories in most trials. As cautious handover motions occurred in only 20% of the trials, it did not adversely affect the overall assembly efficiency too much, and thus, the overall assembly efficiency got a chance to increase. The results also showed that the measurement and display of $T_{R2H}$ in conjunction with the handover operation played a significant role in increasing robot trust in humans (Table 7). The results shown in Table 7 revealed that improvements in HRI, safety, and assembly efficiency for the assembly task involving trust-based handover operation enhanced human trust in the robot as well. Table 10 compares the mean impulse (collision) forces between trust-based and traditional handover conditions. The results showed that the impulse (collision) forces for the trust-based handover condition were almost zero, though there were impulse (collision) forces for the traditional handover condition. Those results clearly showed the superiority of the trust-based handover operation over the traditional handover operation in the collaborative assembly task [42,46].

## 4. Discussion

### 4.1. Interpretation of Trust

We derived the general computational model of trust in Equation (1). We also derived computational models of trust in Equation (2) and Equation (3) to compute human trust in robots and robot trust in humans in real time, respectively. In those equations, the computed trust values were simply the weighted addition (summation) of the performance and faults (mistakes) of the robot and of the human, respectively. Computed trust values do not mean real trust, but these values mean the computational representation of real trust. Computed trust values, as we consider herein, do not represent actual trust; these values merely reveal a sort of artificial trust, which is expected to reflect the mental state of humans and of robots about their collaborators. Performance and faults of an actor or an agent can correlate with the trust of a collaborator or a co-worker about the actor or the agent [73,79]. Research shows that good performance of a worker can generate trust in another interacting co-worker about the worker [73]. Similarly, mistakes or faults made by the worker can adversely affect trust, i.e., it can reduce the trust of an interacting co-worker in the worker [79]. This reduction of trust can lead to a lack of trust or mistrust. Actual trust can be considered as the weighted resultant or sum of trust and lack of trust in real time [73,79].

Computed human trust in robots is artificial, and computed robot trust in humans is more artificial. However, the computed robot trust in humans was considered herein as a means of reflecting the robot’s mental state at the time of collaboration with its human counterpart. Such computed robot trust in humans was used to implement the motion control of the robot’s handover during the assembly task. This effort enhanced the ability of the robot and enabled it to show human-like behaviors. Again, the trust computation models we used herein were deterministic models. Those models could be made more natural or realistic by transforming them into probabilistic or stochastic models. It might be a logical approach to use time-varying or probabilistic values of the real constant coefficients in the models to convert the deterministic models to probabilistic or stochastic models [59]. The trust models we derived in Equations (2) and (3) were computed separately, but they could influence each other. Consequently, the two trust models between the human and the robot could be combined into a single (combined) trust model, which could be termed as “bidirectional or bilateral or mutual trust” [75].

### 4.2. Impact of Latency on Trust Computation, Handover, HRI, and Task Performance

Computation of trust and adjustments of handover configurations of the robot based on computed trust values in real time might result in computational burdens in industrial settings. Latency in the human–robot system could cause delays in trust computation, trust-based message generation, and adjustments of handover configurations based on trust. Such delays might also impact human–robot interaction and system performance [80], because system latency and resulting delays might loosely connect the human co-worker with its robot counterpart via the collaborative system framework. On the other hand, system latency might be helpful for the human co-worker in the sense that it might help the human keep pace with the robot easily. This situation suggests that an optimum level of system latency might be maintained in the human–robot system through investigating performance variations of the system at different latency levels. To do so, the value of *t* (time step) in the trust models could be varied, and its impact on human–robot interaction and system performance could be investigated and analyzed [59].

### 4.3. Sitting Versus Standing Handover

Human-to-robot handover could be performed in a sitting or standing posture of the human [81]. Figure 1 (left) shows the object handover when the human was in a standing posture. However, Figure 1 (right) shows the handover when the human was in a sitting posture. The handover posture (sitting or standing) should be intuitive, and humans should be allowed to decide the posture, considering task situations. In general, a sitting handover posture might be good when the human has high trust in the robot, and thus the human might not need to form an angular (braced) configuration of the handover (Table 3). However, if human trust in the robot went down, and the human needed to create angles or braced configurations to handover an object to the robot, then it might be more convenient for the human to use the standing posture to perform the handover [81].

### 4.4. Comprehensiveness in Evaluations

This article followed the ‘comparison’ strategy as a method to justify the effectiveness of the proposed handover design. The comparison was conducted in three levels: (i) parameter level, (ii) performance level, and (iii) interaction level. If Table 3 parameter values are compared to the results of Table 6, Figure 6, and Table 10, then it becomes clear how the parameter level comparisons were performed between (i) human-to-robot handover and robot-to-human handover, and (ii) the proposed trust-based handover approach and the traditional handover approach. Similarly, Figure 7, Table 7, and Figure 8 show the comparisons of interactions between the proposed novel handover design and the traditional handover design. Table 8 shows comparisons of performance between the proposed novel handover design and the traditional handover design. The evaluation scheme that we used herein to evaluate the effectiveness of the trust-based handover strategies included both objective assessment criteria (e.g., assembly efficiency, handover efficiency, handover success rate, handover safety, impulse forces, etc.) and subjective assessment criteria (e.g., human trust, cognitive workload, engagement, naturalness, transparency, cooperation, etc.) [82,83,84,85]. As we see the presentation of the results, Table 10, Table 8, Table 6, Figure 6, and Table 3 values were objective values. Figure 7, Table 7, and Figure 8 presented subjective assessments. It was realized that the combination of subjective and objective criteria helped make the evaluation scheme complete and comprehensive, which helped cross-check the results [59,82,83,84,85]. Moreover, the evaluation criteria we used were in line with the KPI (key performance indicators) of the human–robot collaborative assembly task [86]. Nonetheless, it might be possible to add more evaluation criteria to the evaluation scheme, and more objective evaluation criteria could be used through innovative sensor applications [82,83,84,85].

### 4.5. Significance, Contribution, and Comparison of the Results

The results presented in experiment 1 clearly demonstrated the human nature and behaviors in terms of kinematics and kinetics in human-to-robot handover of payloads, especially in low-trust situations. The results were novel, and the results clearly created a base for implementing robot-to-human handover strategies based on trust in experiment 2. In experiment 2, the results revealed the effectiveness and efficacy of trust-based handover and assembly task over the traditional assembly and handover task. The evaluation results were comparative, i.e., we compared the evaluation results of trust-based handover and assembly with traditional handover and assembly that did not consider robot trust in humans in adjusting handover configurations and motions [87]. We used the same HRC platform, the same task, and the same subjects in both experiment conditions. As a result, the superiority of the trust-based handover with assembly task over the traditional setup based on the comparative study approach was clear and rational [87].

The main contribution of the paper was that the robot-to-human handover was not fixed or rigid. Instead, the configuration and motion of the robot’s end effector were adjusted during object handover by the robot based on handover situations or conditions (expressed in terms of computed trust values). Such adjustment and flexibility of robot-to-human handover enhanced the overall handover performance, associated assembly task performance, and human-friendliness of the collaborative robot compared to traditional robot-to-human handover when the robot’s handover motion and configuration were fixed or rigid.

We could compare the results presented herein with state-of-the-art results related to collaborative assembly tasks and handovers. However, the state-of-the-art results for human–robot collaborative assembly tasks do not include handover operations, especially robot-to-human handovers of payloads [18,19,20,21,22,23,24]. On the contrary, the state-of-the-art payload handover tasks were not performed in human–robot collaborative assembly settings [33,34,35,36]. As a result, it was difficult to compare the results we obtained herein with the state-of-the-art results. Nonetheless, the handover safety and handover success rate we obtained in this article for trust-based handover clearly proved superiority over the state-of-the-art robot-to-human or human-to-robot handover operations [33,34,35,36]. The overall HRI, assembly task performance, and handover performance obtained herein for the trust-based condition also proved significant in comparison with the state-of-the-art HRC assembly tasks and handover operations [18,19,20,21,22,23,24,33,34,35,36].

### 4.6. Limitations of the Studies

One of the challenging tasks was to measure the angle between the human hand and the robot end effector when the human handed over an object to the robot (Figure 1). The accuracy of angle measurements could impact the determination of braced configurations and handover motions of the robot, due to its low trust in its human counterpart (Figure 2). Furthermore, the configuration and capability of the robot manipulator used in the study could also impact the study and the results. For example, the robot manipulator should be a truly redundant manipulator (having more than 6DOF) [61], which could be used to form the braced configurations more accurately for low-trust situations. It could also be better to use a compliant robot manipulator, which could be safer for the human and which could be bent easily to form the braced configurations [88]. The use of a fast computing device (fast computer or a supercomputer) to process trust computation could make the system more real time-oriented. Even though the number of subjects was decided based on power analysis, the use of more human subjects with diversified backgrounds could further enhance the generality of the results.

### 4.7. Gender of Subjects

Subject’s gender might impact trust computation and trust-based handover decisions [89]. The gender of human subjects used in the experiments might impact human–robot collaborative assembly task performance as well as the evaluation results regarding the effectiveness of trust-based handover [89]. In this article, an equal number of male and female subjects were involved, which minimized such effects. We compared assembly task performance and human–robot interactions between trust-based and traditional handover conditions using the same subjects. Therefore, it is believed that the gender of human subjects either did not impact the comparative results between the two handover conditions or the impacts were minimal. It seems to be logical to test a separate hypothesis to investigate the impacts of subjects’ gender clearly, and adjust trust computation and performance evaluation accordingly [89].

### 4.8. Upgrading to Industry System

The HRC system presented in Figure 1 and Figure 2 may not directly relate to industrial assembly systems employed for human–robot collaborative assembly operations. The presented system is essentially a proof-of-concept system that can serve the purpose of the research presented here, i.e., to investigate the impact of trust-based robot-to-human handover on human–robot collaborative assembly tasks. Nonetheless, the presented system can represent all HRC systems to be used in industries for assembly in manufacturing because the results obtained herein should hold good for all HRC systems, in principle. Real industrial assembly operations may involve tight cycle times, complex quality standards, cost pressures, multi-part coordination, etc. However, all these factors and other relevant factors can be incorporated on top of what we presented herein in the prototype HRC task. When the tasks become complex, subtask allocation between the human and the robot may need to be performed more carefully, addressing task requirements. The time when handover should occur during the collaborative assembly task should be optimized so that it does not impact the assembly cycle and performance significantly. The absolute velocities of handovers and the angles of braced configurations in low-trust situations should be determined considering the task requirements.

A more realistic HRC system can be developed. For example, an HRC system including a handover event can be developed to assemble the center console part of automobiles [59]. In industrial setting, the HRC system (hybrid cell) including the handover operation can be connected to the central automation system (CAS) of the factory by integrating suitable conveyor belts into the HRC system, where one conveyor belt can be used to convey input components to the hybrid cell and another conveyor belt can be used to dispatch the finished products to the next processing unit [36,50]. It is also possible to make the HRC system a part of the central or integrated PLC (programmable logic controller) system of the manufacturing facility [36]. Other advanced facilities and technologies can also be used to augment the abilities and scope of the proposed HRC system, e.g., an integrated and synergistic industry automation system [90], process operation diagrams [91], robot or computer vision systems [92], digital twins technologies [93], etc. The final version of the industrial HRC system can be tested using the actual industry workforce in an industry setting to consider and investigate training requirements, worker acceptance, job displacement concerns, and long-term adaptation effects that may critically influence industrial HRC system success.

## 5. Conclusions and Future Research Directions

We developed a human–robot hybrid cell where a human and a robot collaborated with each other to perform the assembly of multiple parts into a finished/assembled product. We derived a computational model to compute human trust in the robot and robot trust in the collaborating human, and we implemented a real-time trust measurement and display (a human–computer visual interface) scheme in the hybrid cell. We conducted a biomimetic study regarding human-to-robot handover of an object during the collaborative assembly task. The results showed that human psychology was significantly impacted by human trust in the robot, which also impacted the biomechanics of human-to-robot handover. The results revealed that human hand movement slowed down, the angle between the human hand and the robot arm increased, and the human’s grip forces increased if human trust in the robot decreased, and vice versa. Being inspired by those results, we designed a cautious robot-to-human handover strategy based on robot trust in humans. To implement the strategy, we developed a decision-tree type artificial intelligence (AI) algorithm for the handover motion of the robot so that the robot could adjust its handover configurations and motion through kinematic redundancy based on the status of the robot’s trust in the human co-worker in real time during the collaborative assembly task. Objectives of such adjustments in handover configurations and motions were to reduce potential impulse forces due to potential collisions between the robot end effector and the human hand during handover in low robot trust situations and thus increase reliability, safety, overall human–robot interactions, and task efficiency during the assembly task. We then justified the effectiveness of the proposed trust-based assembly and handover motion planning algorithm/strategy for the collaborative assembly task through experimental evaluation. The evaluation results showed that the HRI comprising both pHRI and cHRI, handover success rate, handover safety, assembly efficiency, and human co-worker’s own trust in the collaborating robot improved for the proposed trust-based collaborative assembly and handover motion planning strategy, with a little reduction in the handover efficiency. The results are novel and useful to enhance HRI, safety, and performance in robot-human collaborative tasks involving human–robot handover operations in real-world applications such as flexible lightweight assembly in manufacturing, transportation, logistics, timber processing, disaster management, etc.

In the future, we will develop an innovative motion control algorithm for robot-human handover (e.g., trust-triggered model predictive control) to maximize trust and handover efficiency. We will also implement a reinforcement learning algorithm to learn the optimum trust condition in the collaborative assembly involving handovers. The generality of the results will be further enhanced through increasing the number of human subjects, including a few industry workers (end users), and verifying the results with other types of robotic platforms. We will also validate the results in real industrial scenarios.

## Figures and Tables

**Table 1:** Values of the constant coefficients (trust model parameters) used to compute human trust in the robot in real time.

Parameters (Constant Coefficients)	Values
$b_{1}$	0.439
$b_{2}$	0.094
$c_{1}$	0.368
$c_{2}$	0.099
$q_{1}$	0.00

**Table 2:** Relationship between subjectively assessed and mathematically computed trust values of human co-workers in the robot.

Mean Likert Scale Value Ranges	Mean Computed Value Ranges
5.50–7.0	>0.80–1.0
(High)	(High)
4.0–5.50	0.60–0.80
(Medium)	(Medium)
1.0–4.0	0–0.60
(Low)	(Low)

**Table 3:** Mean human hand velocities, hand angular positions, and grip forces for different computed human trust levels in the robot, and for the first time, handover of an object by the humans to the robot.

Variables	Human Trust in Robot			First Time Handover
	High	Medium	Low	
Mean human hand velocity (m/s)	0.54 (0.02)	0.46 (0.03)	0.32 (0.03)	0.34 (0.04)
Mean human hand angular position (degrees)	1.39 (0.07)	10.63 (0.21)	19.58 (0.69)	19.87 (0.74)
Mean human grip forces (N)	1.62 (0.06)	3.06 (0.14)	5.41 (0.21)	5.64 (0.15)

**Table 4:** Values of the constant coefficients (trust model parameters) used to compute robot trust in the human co-worker in real time.

Parameters (Constant Coefficients)	Values
$\varphi_{1}$	0.402
$\varphi_{2}$	0.126
$\omega_{1}$	0.384
$\omega_{2}$	0.088
$q_{2}$	0.00

**Table 5:** The criteria with description for pHRI assessment.

pHRI Criteria	Description of the Criteria
Transparency	This criterion assessed the quality of displaying contextual information related to human subject’s performance and faults, and the computed robot trust in the human based on performance and faults at different time steps. Such display could make the robot’s mental state transparent to the human subjects as well as make human subjects’ own performance and accuracy transparent to them.
Naturalness	This criterion assessed whether the human subject felt normalcy and whether they realized intuitiveness while collaborating with the robot for the assembly task in the physical environment.
Engagement	This criterion assessed the extent of human subject’s physical involvement with the robot and with the overall collaborative physical system during the collaborative assembly task.
Cooperation	This criterion assessed the level of partnership, sense of working together, and team fluency perceived by the human subject while collaborating with the robot for the assembly task.

**Table 6:** Mean robot arm end effector’s absolute velocities and grip forces for different computed robot trust levels in humans.

Variables	Robot Trust in Humans		
	High	Medium	Low
Mean robot arm’s end effector’s absolute velocity (m/s)	0.52 (0.03)	0.44 (0.02)	0.31 (0.01)
Mean robot arm’s grip forces (N)	1.87 (0.04)	2.18 (0.11)	3.56 (0.16)

**Table 7:** cHRI assessment results between trust-based and traditional handover strategies.

Evaluation Criteria	Handover Conditions	
	Trust-Based	Traditional
Mean overall cognitive workload (%)	26.30 (1.54)	46.21 (2.38)
Mean human trust in the robot	4.84 (0.12)	3.79 (0.14)

**Table 8:** Handover safety, success rate in handover, handover efficiency, and overall assembly efficiency between trust-based and traditional handover conditions.

Evaluation Criteria	Evaluation Results for Assembly and Handover for Two Handover Conditions	
	Trust-Based	Traditional
Handover safety (%)	100	70
Handover success rate (%)	100	70
Mean assembly efficiency (%)	98.68 (2.04)	91.52 (1.67)
Mean handover efficiency (%)	96.94 (1.46)	97.81 (2.41)

**Table 9:** Percentages of different handover trajectories observed for the assembly task with a handover operation based on trust (for trust-based handover condition) for all subjects.

Handover Trajectories	Percentages (%)
Low trust trajectory	5
Medium trust trajectory	15
High trust trajectory	80

**Table 10:** Mean impulse (collision) forces between trust-based and traditional handover conditions.

Evaluation Criteria	Handover Conditions	
	Trust-Based	Traditional
Mean impulse forces (N)	0.01 (0.001)	1.92 (0.09)

## Data Availability

The corresponding author can be contacted for data availability.

## Abbreviations

The following abbreviations are used in this manuscript:

HRI	Human–Robot Interaction
pHRI	Physical Human–Robot Interaction
cHRI	Cognitive Human–Robot Interaction
AI	Artificial Intelligence
GDP	Gross Domestic Product
RQ	Research Question
ROS	Robot Operating System
ANOVA	Analysis of Variance
TLX	Task Load Index
NASA	National Aeronautics and Space Administration
IMU	Inertial Measurement Unit
ARMAV	Autoregressive Moving Average Vector
STD	Standard Deviation
EVA	Extra-Vehicular Activities
HRC	Human–Robot Collaboration
KPI	Key Performance Indicator
PLC	Programmable Logic Controller
DOF	Degree of Freedom
